# Improvement in Heat Stress-Induced Damage to Sperm Quality Following Fecal Microbiota Transplantation from L-Arginine-Treated Mice

**DOI:** 10.3390/ani15060796

**Published:** 2025-03-11

**Authors:** Kai Wang, Yunpeng Suo, Dan Shen, Yifan Shi, Xiaoming Jin, Yansen Li, Chunmei Li

**Affiliations:** Research Centre for Livestock Environmental Control and Smart Production, College of Animal Science and Technology, Nanjing Agricultural University, Nanjing 210095, China; wk@stu.njau.edu.cn (K.W.); 2020105050@stu.njau.edu.cn (Y.S.); t2022067@njau.edu.cn (D.S.); 2020105052@stu.njau.edu.cn (Y.S.); xiaomingjin@njau.edu.cn (X.J.); liyansen@njau.edu.cn (Y.L.)

**Keywords:** L-arginine, heat stress, sperm, testis, microbiota

## Abstract

Heat stress has become a significant concern in animal husbandry, as it adversely affects reproductive performance, particularly sperm quality, through mechanisms that are not fully understood. This study aimed to investigate the protective effects of L-arginine against heat stress-induced sperm damage and explore its potential mechanisms through the modulation of the intestinal microbiota. This study consisted of two experiments. First, in a heat-stressed mouse model, L-arginine was administered to evaluate its effects on the reproductive health of heat-stressed mice. In the second experiment, by transplanting L-arginine-induced changes in the gut microbiota into heat-stressed mice, the protective effects of the microbiota on the sperm of heat-stressed mice were assessed. The findings revealed a significant amelioration of decreased sperm quality and testicular injury induced by heat stress. Post heat stress, mice supplemented with L-arginine presented an increase in seminal vesicle gland weight and index, partial alleviation of testicular tissue morphology, and a substantial increase in testosterone concentration (*p* < 0.05). Additionally, L-arginine upregulated the expression of testosterone synthesis genes and the mRNA levels of sperm generation-related genes, including *3β-HSD*, *Stra8*, *WT1*, and *Gdnf* (*p* < 0.05). Concurrently, L-arginine-induced microbial communities mitigated heat stress-induced decreases in sperm quality and testicular injury, coupled with increases in the mRNA expression levels of *Cyp17a1*, *17β-HSD*, *Plzf*, and *Gdnf* (*p* < 0.05). Furthermore, there was a reduction in the expression of proinflammatory factors, namely, NFκB, *MyD88*, TNF-α, and *TGF-β3* (*p* < 0.05). In conclusion, L-arginine may influence the ratio of beneficial bacteria to harmful bacteria in the intestinal microbiota, thereby reducing inflammation caused by heat stress, maintaining intestinal health, and influencing the microenvironment for spermatogenesis.

## Simple Summary

Heat stress negatively impacts sperm quality, but its underlying mechanisms remain unclear. This study investigated whether L-arginine supplementation could protect against heat stress-induced sperm damage and whether its effects were mediated by gut microbiota. In a mouse model, L-arginine improved sperm quality, testicular health, and testosterone levels while reducing inflammation. Moreover, fecal microbiota transplantation from L-arginine-treated mice also alleviated sperm damage, suggesting a microbiota-mediated protective effect. These findings highlight the potential of gut microbiota modulation in reproductive health under heat stress, providing new insights into microbiota-based strategies for mitigating male infertility in animal husbandry and beyond.

## 1. Introduction

Heat stress, resulting from exposure to elevated temperatures or heat stressors, can exert a multifaceted effect on the male reproductive system. Heat stress significantly influences testicular damage [1]. Elevated temperatures can damage the germ cells of the testes, impeding their functionality. This phenomenon can potentially lead to a reduction in sperm quantity and decreased sperm quality and may even precipitate irreversible testicular injury, consequently affecting male fertility [2]. Interestingly, heat stress may concurrently exert a discernible influence on the gut microbiota [3,4]. Although the gut microbiota may not appear to be directly linked to the reproductive system, research has indicated a close relationship between gut microbiota health and overall well-being [5]. Exposure to high temperatures may trigger physiological stress responses, disrupting the immune system’s balance and the gut microbiota. This may instigate intestinal inflammation or microbial dysbiosis, further impacting overall health, including the reproductive system [6]. Intestinal inflammation and metabolic disturbances can adversely affect testicular function. By modulating gut health, it becomes possible to attenuate inflammation levels and contribute to maintaining normal metabolism and reproductive function [7,8].

L-arginine (L-Arg), a nonessential amino acid, plays a crucial protective role in sperm production under adverse conditions. Previous research has demonstrated its ability to promote sperm formation and maturation in animal models and enhance sperm motility and viability [9,10,11]. In vitro studies have also indicated that L-Arg can increase semen volume and viscosity, improving semen quality [12,13]. Researchers speculate that this protective effect on sperm may stem from its potent antioxidant properties. However, since the inception of research into the gut–testis axis, the gut microbiota status has been significantly associated with sperm development. Consequently, the regulatory role of L-Arg in gut protection and the gut microbiota may have been significantly underestimated. L-Arg can enhance the integrity of the intestinal mucosal barrier, reducing the harm caused by harmful bacteria to the intestinal mucosa and thus safeguarding gut health [14]. It can also regulate the balance of the gut microbiota, promoting the growth of beneficial bacteria while inhibiting the proliferation of harmful bacteria, thereby maintaining the stability of the gut microbiota [15]. Research indicates that L-Arg can increase the population of beneficial bacteria, such as *lactobacilli* and *bifidobacteria*, while reducing the abundance of harmful bacteria, such as *Escherichia coli*, within the intestines [16]. A recent study revealed that L-Arg metabolites inhibit the increase in endotoxins by modulating the abundance of the gut microbiota, further regulating plasma amino acid metabolism to improve semen quality in heat-stressed dogs [17].

The heat stress-induced decline in sperm quality and dysbiosis of the gut microbiota, coupled with the known roles of L-Arg in sperm protection and microbiota modulation, prompted an inquiry into whether L-Arg exerts its protective effect on sperm generation through the gut microbiota. This study aimed to establish a heat stress mouse model and employ fecal microbiota transplantation to investigate whether L-Arg-induced alterations in the gut microbiota can ameliorate sperm damage in mice.

## 2. Materials and Methods

### 2.1. Ethics

The Nanjing Agricultural University Animal Ethics Committee approved all the animal procedures (SYXK(SU) 2022-0031). Forty-two-day-old SPF-grade male ICR mice were purchased from the School of Veterinary Medicine at Nanjing Agricultural University.

### 2.2. Experimental Design and Animal Models

After one week of adaptation, 10 male mice (seven weeks old) were randomly allocated into two groups, with five in each group and were housed in a specific pathogen-free (SPF) facility under strictly controlled conditions. The animal room was maintained at a temperature of 22 ± 2 °C and a relative humidity of 50 ± 10%, with a 12 h light/dark cycle to mimic natural circadian rhythms. Each mouse was housed in a separate cage: a control group—heat stress (Control-HT) and an L-Arg group—heat stress (Arg-HT). L-Arg (Catalog Number (CAS): 74-79-3; purity > 99%, H_2_O solubility: 100 mg/mL, China) and L-alanine (CAS: 56-41-7; purity > 99%, China) were used. L-alanine was added to the drinking water of the control-HT group at a concentration of 7.5 mg/mL (considering the intake protein concentration, we set the concentration of L-alanine to twice that of L-Arg, as the molecular weight of L-Arg is twice that of L-alanine). L-Arg was added to the drinking water of the Arg-HT group at a concentration of 3.75 mg/mL. The L-Arg dosage used in this study was determined on the basis of the effective protection dosage of L-Arg in mice, as reported in the literature [11]. The water was changed every two days during the experiment. All the mice had ad libitum access to drinking water and were housed individually. The water levels in the bottles were monitored daily to ensure that all the mice drank normally. The experiment lasted for 19 days, and 40 min of heat stress (40 ± 1 °C) was applied to the mice daily for 5 days before the end of the experiment. The optimal survival temperature for mice is 20–25 °C. In this experiment, the heat stress temperature was set at 40 ± 1 °C for 40 min each day over a period of 5 consecutive days, which is sufficient to induce heat stress in mice [18,19]. Under normal conditions and heat stress, the daily water intake error of the mice was within 6 mL.

Fecal microbiota transplantation (FMT) mouse model

This study established a murine model for the regulation of the gut microbiota by L-Arg. The model was developed by collecting fecal samples from donor mice under L-Arg treatment and processing these samples to obtain fecal microbiota suspensions. A recipient mouse model was subsequently created for experiments involving oral gavage administration of the fecal microbiota suspensions. These recipient mice were subjected to whole-body heat treatment for five consecutive days on the 94th day of postnatal age. Fecal microbiota transplantation was performed on postnatal day 71 and continued until postnatal day 94, followed by 5 days of heat stress. The mice were euthanized on postnatal day 99. The primary objective of this investigation was to elucidate the mechanisms underlying the ameliorative effects of L-Arg-mediated modulation of the gut microbiota on high-temperature-induced sperm damage.

Donor

The mice were housed and maintained under controlled environmental conditions at a temperature of 22 ± 2 °C and a relative humidity of 60 ± 5%. The mice had ad libitum access to food and water throughout the experimental period. After one week of acclimatization, the mice with similar body weights were randomly allocated into two groups (5 mice/group): the control group and the L-Arg group. L-Arg was added to the drinking water of the Arg group at a concentration of 3.75 mg/mL. To ensure nitrogen balance between the experimental groups, L-alanine was added to the drinking water of the control group at a concentration of 7.5 mg/mL. The water interventions for both groups began when the mice were 71 days old. Fresh fecal samples were collected from the mice in the control and L-Arg groups every two days. The cecal contents were harvested for microbial analysis at 99 days of age, as illustrated in Figure S1. For each sampling event, 200 mg of fresh fecal sample was collected from both the control and L-Arg groups of mice and then suspended in 4 mL of physiological saline. After vortexing for 5 min, the suspensions were centrifuged at 15 g for 3 min. Approximately 3 mL of the supernatant from each sample was collected for subsequent gavage experiments in recipient mice. To ensure the stability of the fecal microbiota in the supernatant, we adhered to the principles of same-day collection, same-day processing, and same-day gavage.

Recipient

After the mice were raised to 70 days of age, those with similar body weights were randomly allocated into two groups, each consisting of 5 mice. Each mouse was housed individually in a separate cage: the control-FMT group received a gavage of 200 µL of the supernatant from the fecal samples processed from the control group), and the Arg-FMT group received a gavage of 200 µL of the supernatant from the fecal samples processed from the L-Arg group. Gavage commenced at 71 days of age and was performed every two days, totaling 14 gavage sessions. Starting at 94 days of age, the mice in both groups underwent daily 40-minute whole-body heat treatment at 40 ± 1 °C for five consecutive days. At 99 days, the mice were euthanized for sampling, as illustrated in Figure S2.

### 2.3. Sampling, Testicular Histology and Sperm Analyses

The testicular tissues were fixed in formaldehyde for 24 h, dehydrated in graded ethanol, embedded in paraffin, and subsequently sectioned to a thickness of 5 μm. These sections were stained with hematoxylin and eosin (HE) and observed under an optical microscope. The cauda epididymides of the mice in the Control-HT and Arg-HT groups were collected and minced in 1 mL of PBS solution. The samples were then incubated at 37 °C for 15 min to release the sperm. After the sample mixture was gently mixed, 10 μL of the incubated sperm was aspirated via a micropipette and placed on a preheated slide at 37 °C. The slides were covered promptly, and the sperm were examined under a 400× microscope objective [20]. The stage temperature was maintained at 37 °C while 3 to 5 fields of view were observed. The density and motility of the semen samples were analyzed via CASA (Spermvision^®^, Version 3.5.6.2; Minitube, Verona, WI, USA).

### 2.4. ELISA

The method utilized in this investigation employed an enzyme-linked immunosorbent assay (ELISA). This technique involves the fixation of known antigens or antibodies onto the bottom surface of a 96-well plate, facilitating the reaction between the enzyme-labeled antigen and the antibody in the solid phase. A mouse testosterone ELISA kit (CAS: ml001948 Shanghai Enzyme Linked Biology, Shanghai, China) from Enzyme Immunoassay Company was used according to the manufacturer’s instructions. The procedure was as follows: Initially, standard solutions were uniformly diluted, and a 96-well plate was prepared with blank, standard, and sample wells. The corresponding standard solutions and samples were added to the designated wells and incubated at 37 °C for 30 min, followed by washing steps. The enzyme conjugate was added to each well and incubated at 37 °C for 30 min. The wells were subsequently washed, and diluted solution A and substrate solution B were added, followed by a 15-min incubation at 37 °C in the dark. Finally, a stop solution was added, and the absorbance was measured at a wavelength of 450 nm. A standard curve was constructed using the absorbance values of standard solutions and their concentrations, enabling the calculation of testosterone content in the samples.

### 2.5. Quantitative Real-Time Polymerase Chain Reaction (qPCR)

A total of 80 mg of testicular tissue stored at −80 °C was placed into a sterile 2 mL zirconium bead tube, and 1 mL of TRIzol reagent was added. The mixture was thoroughly homogenized via a homogenizer. After homogenization, the tube was centrifuged at 800× *g* at 4 °C for 5 min, and the supernatant was transferred to an EP tube. Then, 0.2 mL of chloroform was added. The mixture was vigorously shaken for 15 s to ensure thorough mixing and left at room temperature for 5 min. Afterward, the mixture was centrifuged at 800 × g at 4 °C for 15 min. The upper aqueous phase (approximately 400 ~ 500 µL) was carefully transferred to a new EP tube. To this mixture, 0.75 times the volume of isopropanol was added. The mixture was vigorously shaken for thorough mixing and left at room temperature for 5~10 min. The mixture was subsequently centrifuged at 800× *g* at 4 °C for 10 min. The supernatant was carefully discarded, leaving behind the precipitate. One milliliter of freshly prepared 75% ethanol was added to the precipitate. The precipitate was washed twice by vigorous shaking and centrifuged at 300× *g* at 4 °C for 5 min. The supernatant was discarded, and the pellet was briefly dried in a fume hood for approximately 5 min. Then, 50 μL of DEPC-treated water was added and mixed thoroughly. After extraction, the concentration and purity of the total RNA were determined via a microspectrophotometer, and the samples were diluted to the same concentration. The extracted total RNA was reverse transcribed into cDNA via a reverse transcription kit (CAS: RK26507, ABScript III RT Mix for pPCR with gDNA Remover, ABclonal, Wuhan, China). The reaction conditions were as follows: reverse transcription program: incubation at 37 °C for 2 min, incubation at 55 °C for 15 min, and denaturation at 85 °C for 5 min. The cDNA concentration was measured after reverse transcription, and the samples were stored at −20 °C to avoid repeated freeze‒thaw cycles for subsequent use. The primers used are listed in Supplementary Materials Table S1.

### 2.6. rRNA Sequencing and Analysis

For the fecal samples (n = 5), which were subjected to the gene sequencing procedure, genomic DNA was extracted from all the samples to assess their purity, concentration, and integrity (Methods: NanoDrop 2000 and agarose gel electrophoresis). The V3-V4 hypervariable region was subsequently amplified via primers 338F (5′-ACT CCT ACG GGA GGC AGC AG-3′) and 806R (5′-GGA CTA CHV GGG TWT CTA AT-3′) with an annealing temperature of 55 °C for 27 cycles. On the basis of preliminary testing, the sample results were coded as “A”, indicating the correct band size, appropriate concentration, and suitability for further experimentation. Following successful preliminary testing, the PCR products were used for library construction and subsequently subjected to second-generation sequencing on the gene sequencing platform of Shanghai Majorbio Biopharm Technology Co., Ltd. (Shanghai, China). Sequencing data were analyzed on the Shanghai Majorbio Biopharm Technology Co., Ltd. website platform. The primer sequences are shown in Supplementary Files Table S1. Differences in microbial composition between groups (β diversity) were explored via methods such as principal coordinate analysis (PCoA) and hierarchical clustering. These analyses provided a visual representation of species diversity and group-specific differences.

### 2.7. Statistical Analyses

All endpoints underwent normal distribution validation, and group distinctions were assessed via Student’s t test. Pearson correlation analysis was used to evaluate the relationships between the microbiota and sperm motility, sperm density, and testosterone concentration. Analysis was conducted via GraphPad Prism software, version 8.2.1 (GraphPad Software, Inc., La Jolla, CA, USA), and the data are expressed as the means ± SEMs. Significance was inferred at *p* < 0.05. Highly significance was inferred at *p* < 0.01.

## 3. Results

### 3.1. Changes in Organ Weight and Testicular Tissue Morphology in Mice

After L-Arg was added, the weight and organ index of seminal vesicles in heat-stressed mice increased (*p* = 0.05). Moreover, there was no significant difference in other organ weights or corresponding organ indices (Table 1). After heat treatment, no significant differences in absolute organ weight or indices were detected between the control-FMT and Arg-FMT groups of mice (Table 2).

**Figure 1:**
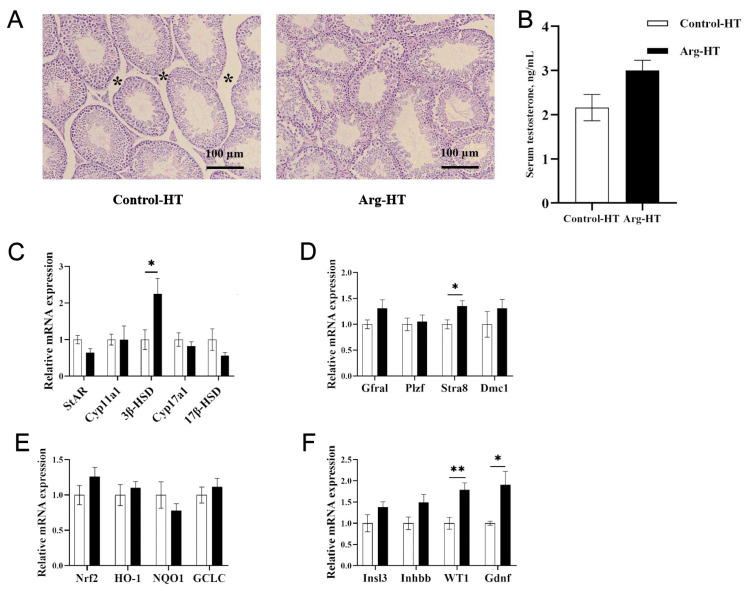
Effects of L-arginine on testicular tissue, testosterone, and related gene expression in heat-stressed mice. (**A**) HE of testicular tissue after heat stress, with * indicating the size of the tight connective tissue between the seminiferous tubules, 100 μm. (**B**) Serum testosterone. (**C**) Testosterone synthesis gene expression. (**D**) Meiosis gene expression. (**E**) Oxidative stress gene expression. (**F**) Spermatogenesis gene expression. n = 5, * represents significant differences, *p* < 0.05. ** represents highly significant differences, *p* < 0.01.

**Figure 2:**
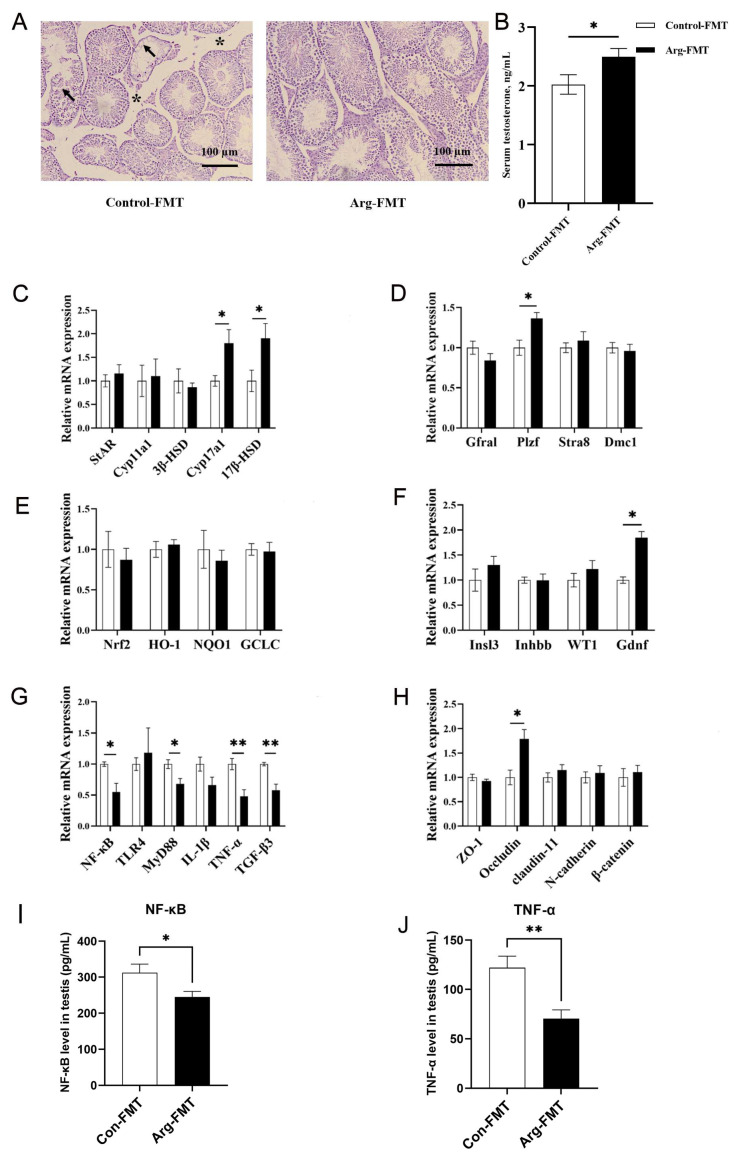
L-arginine affects the gut microbiota in testicular tissue, testosterone, and related gene expression in heat-stressed mice. (**A**) HE of testicular tissue after heat stress, with * indicating the size of the tight connective tissue between the seminiferous tubules, 100 μm. arrows mean vacuolated appearance. (**B**) Serum testosterone. (**C**) Testosterone synthesis gene expression. (**D**) Meiosis gene expression. (**E**) Oxidative stress gene expression. (**F**) Spermatogenesis gene expression. (**G**) Inflammatory gene expression. (**H**) Barrier gene expression. (**I**) NFκB protein abundance in the testis. (**J**) TNF-α protein abundance in the testis. n = 5. * represents significant differences, *p* < 0.05. ** represents highly significant differences, *p* < 0.01.

Following heat stress, the seminiferous tubules in the testes of the mice in the HT control group exhibited atrophy. In contrast, the atrophy symptoms of the seminiferous tubules in the Arg HT group were alleviated (Figure 1A). After heat treatment, the seminiferous tubules in the control-FMT group of mice exhibited extensive atrophy, accompanied by the detachment of numerous germ cells within the seminiferous tubules, resulting in a vacuolated appearance. However, these phenomena were partially alleviated in the Arg-FMT group (Figure 2A).

### 3.2. Changes in Semen Quality and Testosterone Concentration in Gavage-Administered Fecal Microbiota Mice Following Heat Treatment

As shown in Table 3, the addition of L-Arg did not significantly affect the sperm density or rapid sperm movement ratio of heat-stressed mice. However, it improved the total motility and progressive motility of the mice (*p* < 0.05). There was no significant difference in sperm density between the control-FMT and Arg-FMT groups, as shown in Table 4. However, Arg-FMT group mice exhibited greater sperm motility than control-FMT group mice did (*p* < 0.05).

After heat treatment, the serum testosterone concentration in the Arg group of mice was not significantly different from that in the control group (*p* > 0.05), and the serum testosterone concentration in the Arg-FMT group was greater than that in the control-FMT group (*p* < 0.05) (Figure 1B and Figure 2B).

### 3.3. Changes in Reproductive Function-Related Genes in Testicular Tissue of Gavage-Administered Fecal Microbiota Mice Following Heat Treatment

Compared with that in the Control-HT group, the relative expression of the *3β-HSD* gene in the Arg-HT group increased (*p* < 0.05) (Figure 1C). In the context of testosterone biosynthesis-related genes, the expression of the *Cyp17a1* and *17β-HSD* genes in the Arg-FMT group of mice was greater than that in the control-FMT group (*p* < 0.05) (Figure 2C). Compared with that in the HT control group, the expression of the *Stra8* gene was increased in the Arg-HT group (*p* < 0.05) (Figure 1D). In contrast, the expression of the *WT1* and *Gdnf* genes in the Arg-HT group was greater than that in the Control-HT group (*p* < 0.05) (Figure 1F). In terms of genes related to sperm production, the *Plzf* gene was upregulated in the Arg-FMT group compared with the Control-FMT group (*p* < 0.05) (Figure 2D). In the *Nrf2* antioxidant pathway-related genes, there were no changes in gene expression levels in either group of mice (Figure 2E). Compared with that in the control-FMT group, the expression of the *Gdnf* gene, a marker of Leydig cells and supporting cells in the testicular interstitium, was increased in the Arg-FMT group (*p* < 0.05) (Figure 2F). In the *TLR4/NF-κB* inflammatory pathway, the gene expression levels of *NF-κB*, *MyD88*, *TNF-α*, and *TGF-β3* in the Arg-FMT group of mice were lower than those in the Control-FMT group (*p* < 0.05) (Figure 2G) The ARG-induced microbiota significantly attenuated the elevated protein concentrations of NFκB and TNF-α induced by heat stress (Figure 2I,J).

### 3.4. Effects of L-Arg on the Gut Microbiota of Heat-Stressed Mice

**Figure 3:**
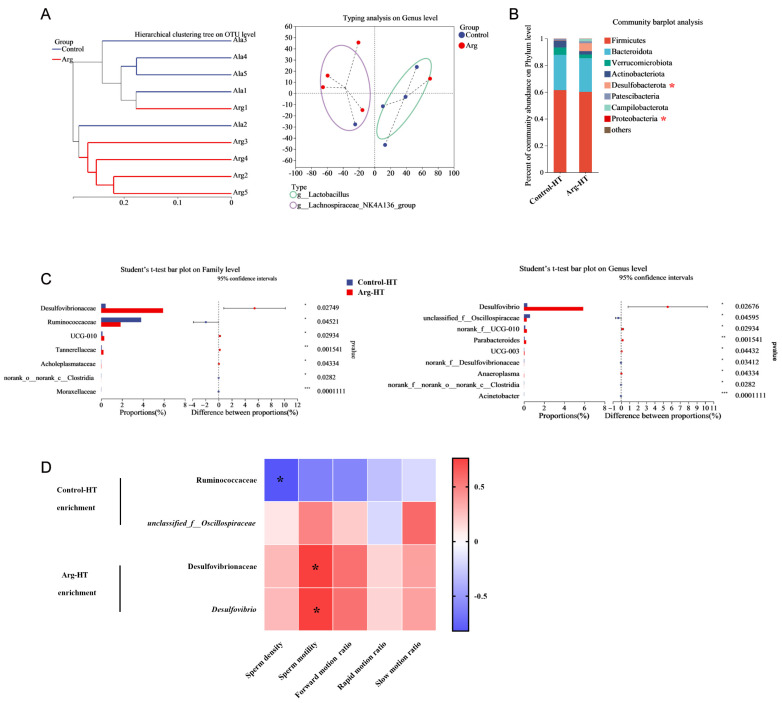
Effects of L-arginine on the gut microbiota of heat-stressed mice. (**A**) Hierarchical clustering tree at the OTU level and typing analysis at the genus level. (**B**) Community bar plot analysis. (**C**) Differential bacterial screening. (**D**) Correlation between sperm quality and bacteria. n = 5 * represents significant differences, *p* < 0.05. ** represents significant differences, *p* < 0.01. *** represents significant differences, *p* < 0.001.

Analysis of the intestinal microbiota in the two groups of mice revealed distinct separation trends in the PCoA plot, as shown in Figure 3A. Specifically, the Control-HT and Arg-HT groups clearly separated, with the primary differences concentrated along the PC1 axis (30.2%). Both groups were predominantly composed of Firmicutes, Bacteroidota, Verrucomicrobiota, and Actinobacteria at the phylum level. T test analysis revealed an increase in the abundance of *Desulfobacterota* in the intestinal microbiota of the Arg-HT group compared with the Control-HT group (*p* < 0.05). Moreover, the abundance of *Proteobacteria* was lower in the Arg-HT group than in the Control-HT group (*p* < 0.05), as shown in Figure 3B. Further analysis at the family level revealed significant enrichment of *Ruminococcaceae*, *norank_o__norank_c__Clostridia*, and *Moraxellaceae* in the Control-HT group (*p* < 0.05). In contrast, *Desulfovibrionaceae*, *UCG-010*, *Tannerellaceae*, and *Acholeplasmataceae* were enriched in the Arg-HT group (*p* < 0.05), as illustrated in Figure 3C. T test analysis at the genus level revealed enrichment of *unclassified_f__Oscillospiraceae*, *norank_f__Desulfovibrionaceae*, *norank_f__norank_o__norank_c__Clostridia*, and *Acinetobacter* in the Control-HT group (*p* < 0.05). Moreover, *Desulfovibrio*, *norank_f__UCG-010*, *Parabacteroides*, *UCG-003*, and *Anaeroplasma* were enriched in the Arg-HT group (*p* < 0.05).

### 3.5. Correlation Analysis Between Differential Gut Microbiota and Semen Quality

Correlation analysis between differential microbiota and semen quality in mice revealed significant associations. Specifically, *Ruminococcaceae* was negatively correlated with sperm density (*p* < 0.05). Moreover, *Desulfovibrionaceae* and *Desulfovibrio* were positively correlated with semen quality (Figure 3D).

### 3.6. Changes in the Gut Microbiota Composition of Mice with Gavage-Administered Fecal Microbiota Following Heat Treatment

**Figure 4:**
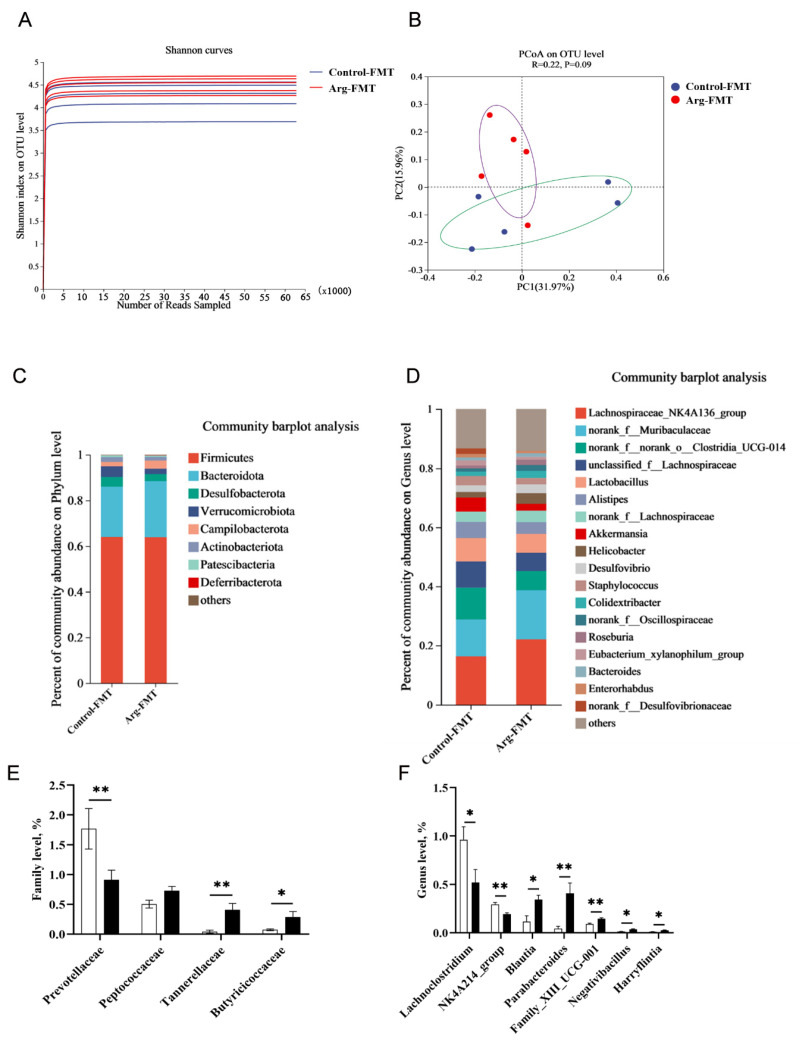
Effects of L-arginine-induced gut microbiota transplantation on the gut microbiota of heat-stressed mice. (**A**) α Diversity. (**B**) β Diversity. (**C**) Community bar plot analysis at the phylum level. (**D**) Community bar plot analysis at the genus level. (**E**) Differential bacterial screening at the family level. (**F**) Differential bacterial screening at the genus level. n = 5. * represents significant differences, *p* < 0.05. ** represents highly significant differences, *p* < 0.01.

The dilution curve plot revealed that the majority of species diversity in the Arg-FMT group was greater than that in the control-FMT group (Figure 4A). The PCoA plot revealed a trend toward separation in the gut microbiota of the mice between the Arg-FMT and control-FMT groups, with the main differences concentrated along the PC1 axis (31.97%) (Figure 4B).

Further analysis of the species composition at the phylum level revealed that both groups of mice were dominated by four major phyla: Firmicutes, Bacteroidota, Desulfobacterota, and Verrucomicrobiota (Figure 4C). At the genus level, the relative abundances of *Lachnospiraceae_NK4A136_group* and *norank_f__Muribaculaceae* were significantly greater in the Arg-FMT group than in the Control-FMT group. In comparison, the abundances of *norank_f__norank_o__Clostridia_UCG-010*, *unclassified_f__Lachnospiraceae, Lactobacillus*, *Alistipes*, and *Akkermansia* were significantly lower in the Arg-FMT group (Figure 4D).

T test analysis at the order level revealed that the abundance of Prevotellaceae was significantly lower in the Arg-FMT group than in the Control-FMT group (*p* < 0.05). In comparison, the abundances of *Peptococcaceae*, *Tannerellaceae*, and *Butyricicoccaceae* were significantly greater in the Arg-FMT group, with differences in *Tannerellaceae* and *Butyricicoccaceae* (*p* < 0.05) (Figure 4E). At the genus level, the abundances of *Lachnoclostridium and NK4A214_group* were lower in the Arg-FMT group than in the Control-FMT group (*p* < 0.05). In comparison, the abundances of *Blautia*, *Parabacteroides*, *Family_XIII_UCG-001*, *Negativibacillus*, and *Harryflintia* were greater in the Arg-FMT group (*p* < 0.05) (Figure 4D).

## 4. Discussion

Heat stress, characterized by exposure to elevated temperatures, harms testicular function and sperm quality, posing a significant threat to male reproductive health [21,22]. This study investigated the potential protective role of L-Arg in mitigating heat stress-induced sperm damage through regulating the intestinal microbiota. Comprehensive analysis through fecal microbiota transplantation revealed intricate interactions among L-Arg, the intestinal microbiota, and semen quality. Our findings demonstrate that L-Arg significantly ameliorates the decline in sperm quality and dysbiosis of the intestinal microbiota induced by heat stress. This effect may be attributed to the modulation of the microbial balance in the gut, which promotes beneficial bacterial growth and inhibits harmful bacterial proliferation. These actions collectively contribute to maintaining intestinal health, consequently impacting the environment for sperm production.

The assessment of organs and relative weight is frequently employed to evaluate the developmental status of organs and the entire organism [23]. Research has revealed a significant reduction in the weight of reproductive glands under heat stress conditions [24]. In this study, following exposure to heat stress, the weight and organ index of the seminal vesicle glands supplemented with Arg were notably greater than those in the control group. The seminal vesicle gland, which serves as an accessory gland in male animals, contributes 60~70% of the nutrients in seminal plasma [25]. Consequently, L-Arg has an ameliorative effect on the reproductive glands of mice under high-temperature conditions. However, though transplantation of L-Arg-induced changes in the gut microbiota, no significant differences in the weights or indices of organs, such as the seminal vesicle gland, were detected. This may be potentially associated with the duration of treatment.

In this study, we observed that L-Arg significantly altered the gut microbiota composition in mice. In L-Arg-treated mice, there was a substantial increase in the abundance of beneficial bacteria and a marked decrease in the abundance of harmful bacteria. Our investigation of the gut microbiota composition following L-Arg supplementation revealed significant changes, particularly in the abundance of specific bacterial taxa. L-Arg administration resulted in a significant reduction in *Deferribacteraceae NK4A136 group* abundance and a shift toward dominance by the *Helicobacteraceae_NK4A136* group. These alterations in gut microbiota diversity and composition suggest a potential role for L-Arg in modulating the intestinal microbiota under heat stress conditions. Early studies established its ability to promote sperm formation and maturation, increase sperm motility, and improve sperm viability in animal models [9,26,27,28]. In vitro investigations further suggest that L-Arg augments semen volume and viscosity, enhancing semen quality [29,30]. Furthermore, our findings indicate that L-Arg significantly improves sperm motility, the forward sperm movement ratio, and the slow sperm movement ratio in heat-stressed mice. Additionally, transplantation of the L-Arg-induced microbiota similarly resulted in a significant increase in sperm quality.

The seminiferous tubules, which harbor many germ cells, serve as sites for sperm differentiation [31]. Elevated temperatures reduce the diameter of seminiferous tubules in mice, leading to germ cell apoptosis, and, following heat stress, sperm quality decreases, and testosterone levels decrease. Histological examinations revealed conspicuous atrophy of convoluted seminiferous tubules in heat-stressed mice, indicative of compromised testicular structure and impaired spermatogenesis. L-Arg supplementation mitigates testicular atrophy, suggesting its potential protective role against heat-induced testicular injury. Following the induction of the gut microbiota by L-Arg, testicular tissue damage in heat-stressed mice is similarly alleviated. Furthermore, testosterone, a pivotal male sex hormone predominantly secreted by Leydig cells, stimulates spermatogenesis [32]. Studies indicate that elevated temperatures impact Leydig cell secretory function, downregulating the expression of steroidogenic enzyme-encoding genes and reducing testosterone production [33]. Both L-Arg and its regulated microbiota significantly increase testosterone concentrations in heat-stressed mice. These findings underscore the ability of L-Arg and its ability to modulate the microbiota to increase testicular tissue integrity, improve sperm viability, and increase testosterone concentrations, thereby positively impacting male reproductive function under stressful conditions.

Spermatogenesis is a complex process regulated by numerous genes throughout continuous differentiation and division. The addition of L-Arg upregulated *Stra8* gene expression in heat-stressed mice in this study. *Stra8*, a key regulator of spermatogonial cell meiotic division, causes severe defects in sperm function when it is deficient in male mice, suggesting that L-Arg upregulates the gene expression of *Star8* under heat stress conditions to ensure normal spermatogenesis in mice exposed to elevated temperatures. Studies indicate that dietary supplementation with L-Arg increases the expression of *Nrf2* antioxidant pathway-related genes in heat-stressed mice. In this study, the addition of L-Arg had a specific effect on *Nrf2* pathway-related genes in heat-stressed mice, although the effect was not statistically significant, possibly because of considerable individual variations within the group, warranting further validation. Sertiative cells and interstitial cells play a supportive and protective role in spermatogenesis. *WT1* and *Gdnf* are active factors in testicular interstitial and supportive cells [34,35,36,37]. In this study, the gene expression of *WT1* and *Gdnf* was significantly upregulated after the addition of L-Arg in heat-stressed mice. *WT1* is reportedly involved in the positioning and counting of supporting cells, with its deficiency leading to a compromised blood‒testis barrier and germ-cell death. Additionally, the neurotrophic factor *Gdnf* secreted by supporting cells regulates the proliferation and differentiation of spermatogonial stem cells, promoting sperm production. Therefore, L-Arg may protect against spermatogenesis by upregulating the expression of the meiotic division regulator Star8 and preserving the secretory function of testicular supporting cells. FMT from L-Arg-treated recipient mice subjected to heat stress and heat stress alleviation significantly improved sperm quality and the expression of reproduction-related genes. Furthermore, our study revealed significant upregulation of the steroidogenic enzymes *Cyp17a1* and *17β-HSD* in heat-stressed mice treated with FMT. These enzymes are essential for the production of androgens, glucocorticoids, and testosterone, which are crucial for spermatogenesis and sperm cell maturation [38]. Additionally, the transcription factor *Plzf*, which regulates the self-renewal of spermatogonial stem cells, showed increased expression, indicating a supportive environment for continuous sperm production [39,40]. The inflammatory response pathways involving *NFκB* and *MyD88* were also evaluated. FMT resulted in the downregulation of NFκB and *MyD88*, suggesting a protective effect against inflammatory damage and a reduced innate immune response in reproductive tissues. Furthermore, TNF-α was significantly downregulated, indicating reduced inflammatory stress. In contrast, the expression of *TGF-β3*, which is known for its role in immune tolerance, was downregulated, suggesting that it plays a role in maintaining testicular health under heat stress conditions [41]. FMT from L-Arg-treated, heat-stressed mice significantly improved sperm quality and the expression of reproductive- and inflammation-related genes, indicating the potential of L-Arg-treated donor microbiota to ameliorate heat stress-induced sperm damage.

In this study, the effective absorption of L-Arg may have led to an increased testosterone concentration in the FMT experiment, potentially affecting the experimental results. The testosterone levels in L-Arg-treated mice were greater than those in control mice, which makes distinguishing the independent effects of elevated testosterone from those of FMT in the experimental design difficult. Therefore, we explicitly state that the potential confounding effect of testosterone in FMT or the role of microbes in FMT may be responsible for the observed ameliorative effects. Despite the potential confounding factors mentioned above, we observed significant improvements in sperm quality and testicular function in the L-Arg-FMT treatment group. Given that the testosterone content in fecal matter is too low to have a direct effect, L-Arg may exert protective effects by modulating the intestinal microbiota, independent of changes in testosterone levels. The absence of in vitro fertilization and in vitro culture experiments to directly assess the fertilization potential of treated sperm. While our findings indicate improvements in sperm quality and related molecular markers, future studies should include functional assays to confirm their impact on embryo development.

## 5. Conclusions

By integrating the findings from the present study, we confirmed the potential role of L-Arg in ameliorating sperm damage induced by heat stress. This protective effect is likely realized through its ability to modulate the intestinal microbiota, promote the growth of beneficial bacteria, suppress the proliferation of harmful bacteria, maintain intestinal health, and consequently positively impact testicular tissue integrity and the environment for sperm production. These results underscore the potential of L-Arg as a promising therapeutic approach to help preserve male reproductive function under adverse environmental conditions. Nevertheless, this study has several limitations, such as the need for further investigation into the specific mechanisms by which L-Arg modulates the intestinal microbiota to improve sperm damage. Future research should delve deeper into these mechanisms to better comprehend the potential therapeutic value of L-Arg in maintaining reproductive system health and the intestinal microbiota balance under challenging conditions.

## Figures and Tables

**Table 1:** Effects of L-Arg supplementation on organ weight in heat-treated mice.

	Control-HT	Arg-HT	*p*
Seminal vesicle gland, g	0.30 ± 0.04 ^b^	0.42 ± 0.03 ^a^	0.05
Seminal vesicle gland organ index, %	0.79 ± 0.10 ^b^	1.07 ± 0.07 ^a^	0.05
Epididymis, g	0.12 ± 0.02	0.11 ± 0.01	0.74
Epididymis organ index, %	0.30 ± 0.04	0.28 ± 0.02	0.56
Testicles, g	0.29 ± 0.03	0.28 ± 0.01	0.84
Testicular organ index, %	0.75 ± 0.15	0.71 ± 0.04	0.62

Note: Organ index = organ weight (g)/body weight (g) × 100. control group—heat stress (Control-HT) and an L-Arg group—heat stress (Arg-HT). The superscript letters a and b represent significant differences. n = 5.

**Table 2:** Changes in organ weight after heat stress in fecal microbiota-transplanted mice.

	Control-FMT	Arg-FMT	*p*
Seminal vesicle gland, g	0.37 ± 0.03	0.35 ± 0.03	0.58
Seminal vesicle gland organ index, %	0.92 ± 0.09	0.85 ± 0.08	0.55
Epididymis, g	0.12 ± 0.01	0.11 ± 0.01	0.35
Epididymis organ index, %	0.28 ± 0.01	0.28 ± 0.01	0.68
Testicles, g	0.23 ± 0.03	0.29 ± 0.01	0.10
Testicular organ index, %	0.61 ± 0.09	0.72 ± 0.03	0.27

Note: Organ index = organ weight (g)/body weight (g) × 100. FMT: Fecal microbiota transplantation, n = 5.

**Table 3:** Effects of L-Arg supplementation on the semen quality of heat-treated mice.

Semen Quality	Control-HT	Arg-HT	*p*
Sperm density, 10^7^/mL	0.87 ± 0.17	1.02 ± 0.15	0.55
Total motility, %	49.40 ± 2.70 ^b^	63.55 ± 3.32 ^a^	<0.01
Progressive motility, %	41.49 ± 2.83 ^b^	57.46 ± 3.43 ^a^	<0.01

Note: The forward movement ratio, fast movement ratio, and slow movement ratio represent the motility characteristics of the sperm, n = 5, different letters represent significant differences *p* < 0.05.

**Table 4:** Changes in semen quality after heat stress in fecal microbiota-transplanted mice.

Semen Quality Parameters	Control-FMT	Arg-FMT	*p*
Sperm density, 10^7^/mL	3.90 ± 0.19	3.38 ± 0.31	0.15
Total motility, %	22.09 ± 2.09 ^b^	40.71 ± 3.43 ^a^	< 0.01
Progressive motility, %	15.94 ± 1.78 ^b^	33.42 ± 3.58 ^a^	< 0.01

Note: The forward movement ratio, fast movement ratio, and slow movement ratio represent the motility characteristics of the sperm, n = 5, different letters represent significant differences *p* < 0.05.

## Data Availability

All the data can be found in the manuscript or in the Supplementary Materials. Microbiome data were deposited in the US National Center for Biotechnology Information (NCBI). No. PRJNA1060859.

## Supplementary Materials

The following supporting information can be downloaded at: https://www.mdpi.com/article/10.3390/ani15060796/s1, Figure S1: Collection process of donor microbiota; Figure S2: Fecal microbiota transplantation; Table S1: Primers used for real-time PCR.
