# Supplementary material for: Improvement in Heat Stress-Induced Damage to Sperm Quality Following Fecal Microbiota Transplantation from L-Arginine-Treated Mice
Source: Animals (Basel). 2025 Mar 11;15(6):796. doi: 10.3390/ani15060796 (PMC11939313; doi:10.3390/ani15060796)
Supplement: Supplementary file 1 [file animals-15-00796-s001.zip › animals-3508278-supplementary.docx]

Figure S1 Collection process of donor microbiota


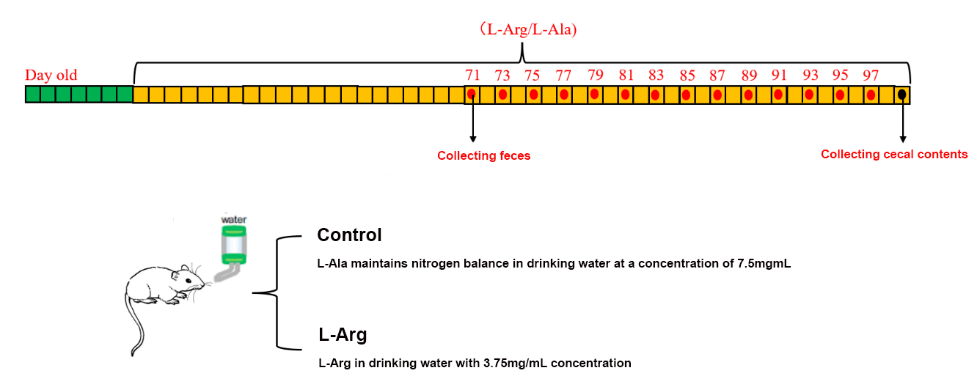


Figure S2 Fecal microbiota transplantation


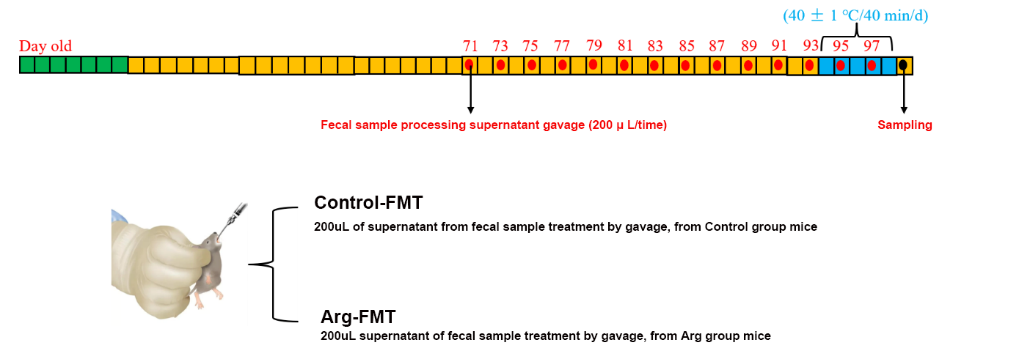


Table S1 Primers used for real-time PCR

| Gene | Sequence (5’to 3’) | Size (bp) |
| --- | --- | --- |
| *β-actin* | F: CTAAGGCCAACCGTGAAAAGAT  R: CACAGCCTGGATGGCTACGT | 83 |
| *StAR* | F: CGGGTGGATGGGTCAAGTTC  R: GCACTTCGTCCCCGTTCTC | 188 |
| *Cyp11a1* | F: AGGTCCTTCAATGAGATCCCTT  R: TCCCTGTAAATGGGGCCATAC | 137 |
| *3β-HSD* | F: AGCTCTGGACAAAGTATTCCGA  R: GCCTCCAATAGGTTCTGGGT | 234 |
| *Cyp17a1* | F: GTCGCCTTTGCGGATAGTAGT  R: TGAGTTGGCTTCCTGACATATCA | 120 |
| *17β-HSD* | F: TTAGTCGGACACTGGAAAAGC  R: ATTCTGGCTCTCACCGGAAGT | 224 |
| *Gfra1* | F: GTGGCAATGACCTGGAAGAT  R: ATTGCCAAAGGCTTGAATTG | 89 |
| *Plzf* | F: AAACGGTTCCTGGACAGTTTGCGAC  R: CCAGTATGGGTCTGTCTGTGTGTCTCC | 140 |
| *Stra8* | F: GTTTCCTGCGTGTTCCACAAG  R: CACCCGAGGCTCAAGCTTC | 151 |
| *Dmc1* | F: CCATATCACTACTGGGAGC  R: GTACTGCTTCATGGTCTAC | 89 |
| *Nrf2* | F: CAGTGCTCCTATGCGTGAA  R: GCGGCTTGAATGTTTGTC | 109 |
| *HO-1* | F: ACAGATGGCGTCACTTCG  R: TGAGGACCCACTGGAGGA | 128 |
| *NQO1* | F: CTTTAGGGTCGTCTTGGC  R: CAATCAGGGCTCTTCTCG | 102 |
| *GCLC* | F: GGATGATGCCAACGAGTC  R: GTGAGCAGTACCACGAATA | 180 |
| *Insl3* | F: ACTGATGCTCCTGGCTCTGG  R: GGAGATGTCTCTGCTCTAGC | 194 |
| *Inhbb* | F: GGTCCGCCTGTACTTCTTCGTCT  R: GGTATGCCAGCCGCTACGTT | 217 |
| *WT1* | F: ATCCCAGGCAGGAAAGTGTG  R: GTGCTGTCTTGGAAGTCGGA | 111 |
| *Gdnf* | F: CGCTGACCAGTGACTCCAATA  R: AGTTAAAACGCACCCCCGAT | 227 |
